# Correlationship between Ki67, VEGF, and p53 and Hepatocellular Carcinoma Recurrence in Liver Transplant Patients

**DOI:** 10.1155/2021/6651397

**Published:** 2021-04-15

**Authors:** Xia Zhang, Zhixian Wu, Yonghai Peng, Dongliang Li, Yi Jiang, Fan Pan, Yi Li, Yanhua Lai, Zhongyuan Cui, Kun Zhang

**Affiliations:** ^1^Department of Hepatobiliary Disease, The 900th Hospital of the People's Liberation Army Joint Service Support Force (Dongfang Hospital), Xiamen University, Fuzhou, Fujian, China; ^2^Department of Oncology, The 900th Hospital of the People's Liberation Army Joint Service Support Force (Dongfang Hospital), Xiamen University, Fuzhou, Fujian, China; ^3^Department of Hepatobiliary Surgery, The 900th Hospital of the People's Liberation Army Joint Service Support Force, China; ^4^Department of Oncology, 920th Hospital of Joint Logistics Support Force, China; ^5^Department of Transplantation, People's Hospital of Guangxi Zhuang Autonomous Region, China; ^6^Department of Hepatobiliary Surgery, Xiang'an Hospital, Xiamen University, Xiamen, China

## Abstract

**Background and Aims:**

Patients with hepatocellular carcinoma (HCC) who undergo orthotopic liver transplantation (OLT) are at risk for posttransplant tumor recurrence. The aim of this study was to evaluate the correlation between the expression of Ki67, VEGF, and p53 in HCC and clinicopathological characteristics of HCC patients, as well as their predictive value for HCC recurrence after OLT.

**Methods:**

60 patients who underwent OLT and were found to have HCC in the liver explant. The expression of Ki67, VEGF, and p53 in HCC was detected by immunohistochemistry.

**Results:**

Ki67 was associated with the tumor number and the grade of differentiation at baseline. VEGF was associated with the diameter and number of tumors, tumor differentiation, and lymph node metastasis. p53 was associated with the tumor diameter and tumor encapsulation. The expression of Ki67, VEGF, and p53 in HCC was correlated with the tumor recurrence after OLT, respectively. Among them, VEGF was an independent predictor for tumor recurrence after OLT.

**Conclusion:**

Ki67, VEGF, and p53 are associated with the recurrence of HCC after OLT. VEGF independently predicts the recurrence of HCC.

## 1. Introduction

Hepatocellular carcinoma (HCC) is one of the leading causes of cancer-related death globally [1, 2]. According to statistics, the annual mortality of HCC in China accounts for 55% of the global one. Because the initial symptoms of HCC are not evident, the patients are often in the intermediate or advanced stages at diagnosis. The survival of untreated HCC patients after diagnosis is usually less than 6 months [3, 4].

To date, orthotopic liver transplantation (OLT) is one of the best options in case of end-stage liver disease [5]. With the advances in medicinal treatment, five-year survival rates after OLT of over 75% have been widely observed [6, 7]. Although the survival rate is relatively high, the risk of recurrence is the major concern in transplanted patients. Clinical factors that are related to the recurrence after OLT include the size and number of tumors, micro/macrovascular invasion, and high levels of serum alpha-fetoprotein (AFP) [8].

Tumor-related factors including Ki67, VEGF, and p53 have been reported to play a role in the development and progression of HCC [9–11].

Tumor proliferating antigen (Ki67) is a nuclear protein that is recognized as a sensitive marker for cell proliferation [11]. Ki67 is highly expressed in numerous human solid tumors and is correlated with patient prognosis. Several studies found that the expression of Ki67 in numerous cancers including prostate cancer, lung cancer, and HCC was negatively correlated with the therapeutic efficacy and prognosis [12–14]. However, the predictive value of Ki67 in the recurrence of HCC after OLT remains unclear.

Vascular endothelial growth factor (VEGF) is an important factor that mediates angiogenesis. It plays an indispensable role in tumor growth, invasion, and metastasis, as well as patient prognosis [15]. Nagoshi showed that VEGF promoted the proliferation of tumor vascular endothelial cells and served as an early marker of angiogenesis in HCC [16]. In a study of human OLT, it was suggested that the high recurrence rate after transplantation was associated with the expression of VEGF in liver grafts during the acute rejection phase [17]. Although high expression of VEGF promoted the recurrence of HCC after transplantation, whether VEGF predicts the recurrence remains unclear.

*p53* is a tumor suppressor gene that regulates cell cycle and apoptosis [9]. Its mutations were found to be tumorigenic in several cancers including colon and prostate cancers, and HCC [18–20]. A study showed that the rates of gene mutation and upregulated expression of *p53* in HCC patients were 31.5% and 35.0%, respectively, and the expression of p53 was significantly associated with the poor prognosis of HCC patients [21]. However, whether p53 is associated with the recurrence of HCC after OLT has not been revealed.

Detection of factors that are associated with HCC recurrence may predict the tumor recurrence in HCC patients after OLT. In this study, we detected the expression of Ki67, VEGF, and p53 in HCC by immunohistochemical staining and explored their predictive value in tumor recurrence after OLT.

## 2. Materials and Method

### 2.1. Study Population Clinicopathological Characteristics

The study population included 60 patients who had undergone OLT at Dongfang Hospital and had complete follow-up data. All patients were diagnosed of HCC before OLT. Recurrent HCC were confirmed by a liver biopsy and pathological examination.

### 2.2. Clinicopathological Parameters of HCC Patients

The characteristics of HCC patients including the Child-Pugh class, the diameter and number of tumors, serum level of AFP, TNM stage, vascular invasion, liver cirrhosis, tumor encapsulation, lymph node metastasis, and tumor differentiation were collected. Patients with recurrence of HCC within 2 years were divided into the recurrence group (*n* = 37). Patients whose HCC recurred after 2 years or did not recur until the last visit were divided into the control group (*n* = 23).

### 2.3. Immunohistochemical Staining

Five-*μ*m slices were obtained from paraffin-embedded specimens of tumor. Sections were dewaxed in xylene and rehydrated in alcohol followed by wet autoclave pretreatment (10 minutes at 120°C) in citrate buffer for antigen retrieval. These were rinsed in phosphate-buffered saline. Immunohistochemical staining for antibodies to Ki67 (Cat: ab15580, Abcam, Cambridge, MA), VEGF (Cat: 19003-1-AP, Proteintech, Chicago, USA), and p53 (Cat: ab1101, Abcam, Cambridge, MA) was performed using the avidin-biotin-peroxidase complex method. The primary antibody was applied to the sections and allowed to react for 25 min at room temperature. The sections were then incubated with biotinylated anti-mouse/rabbit antibody (1 : 100 dilution for Ki67, VEGF, and p53) for 25 min and avidin-biotin-peroxidase reagent for 25 min. After color development with diaminobenzimide, the sections were counterstained with hematoxylin.

### 2.4. Statistical Analysis

The correlation between Ki67, VEGF, and p53 and clinicopathological characteristics were analyzed by the *χ*^2^ test and Fisher test. The correlation between Ki67, VEGF, and p53 and tumor recurrence after OLT were analyzed by single factor survival analysis and COX multivariate regression. A *P* value of <0.05 was considered statistically significant. All statistical analyses were performed using the SPSS 19.0 analysis software.

## 3. Results

The patients were three women and 57 men with a mean age of 55 ± 15 years. 54 patients were positive for hepatitis B surface antigen (HBsAg), and six were negative. 31 patients were with a tumor diameter of <5 cm, and 29 were of 5-15 cm. The AFP level was ≥400 *μ*g/L in 20 patients and <400 *μ*g/L in 40. Postoperative pathological examination showed 11 cases of poorly differentiated HCC, 37 of moderately differentiated HCC, and 12 of well-differentiated HCC. The recurrence of tumors included eight cases of intrahepatic recurrence, seven of intrahepatic and extrahepatic recurrence, 18 of lung metastasis, nine of bone metastasis, six of lymph node metastasis, and three of brain metastasis.

### 3.1. Correlationship between Ki67, VEGF, and p53 and Clinicopathological Characteristics of HCC Patients

The expression of Ki67 was correlated with the number of tumors (*P* = 0.005) and the grade of tumor differentiation (*P* = 0.038). However, it was not associated with other clinicopathological characteristics of HCC patients (Table 1). The expression of VEGF was associated with the diameter and number of tumors (*P* = 0.037 and *P* = 0.005), tumor differentiation (*P* = 0.035), and lymph node metastasis (*P* = 0.025), whereas it was not related to other clinicopathological characteristics (Table 1). The expression of p53 was correlated with the tumor diameter (*P* = 0.044) and tumor encapsulation (*P* = 0.022). There was no correlation between p53 and other clinicopathological characteristics (Table 1).

### 3.2. The Expression of Ki67, VEGF, and p53 in HCC

**Figure 1:**
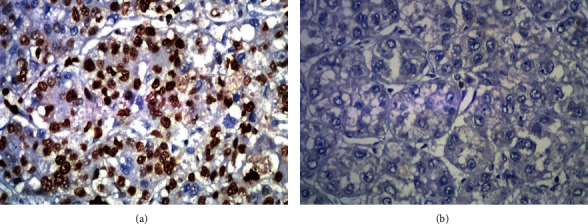
(a) Positive immunostaining with nuclear Ki67 expression in HCC. (b) Negative Ki67 expression with low nuclear reactivity in HCC.

**Figure 2:**
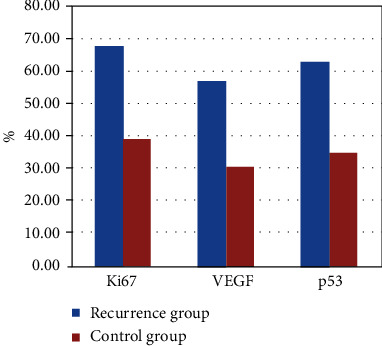
The positive expression rates of Ki67, VEGF, and p53 in the recurrence group and the control group.

**Figure 3:**
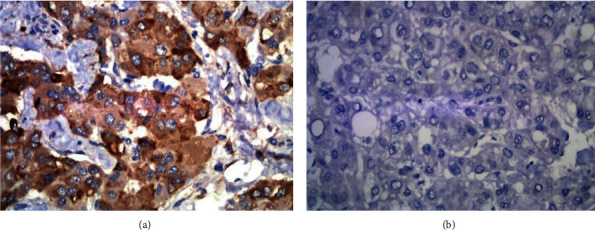
(a) Positive immunostaining with VEGF expression in HCC. (b) Negative VEGF expression immunostaining in HCC.

**Figure 4:**
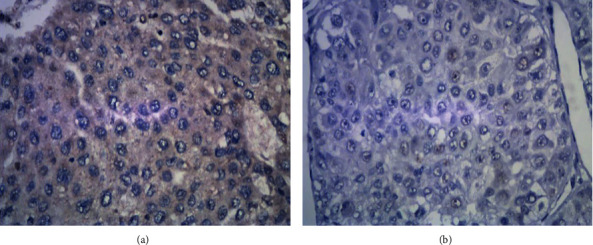
(a) Positive immunostaining with p53 expression in HCC. (b) Negative p53 expression immunostaining in HCC.

In the postoperative HCC samples from 37 patients in the recurrence group and 23 in the control group, the positive expression rates of Ki67 were 67.5% (25/37) and 39.1% (9/23), respectively (Figures 1(a), 1(b), and 2). The positive expression rates of VEGF were 56.7% (21/37) and 30.4% (7/23), respectively (Figures 3(a), 3(b), and 2). The positive expression rates of p53 were 62.1% (23/37) and 34.7% (8/23), respectively (Figures 4(a), 4(b), and 2). Logistic single-factor statistical analysis showed that the positive expression rates of Ki67, VEGF, and p53 were different between the recurrence group and the control group, respectively (*P* = 0.015, 0.008, and 0.035, Table 2).

### 3.3. Correlationship between the Expression of Ki67, VEGF, and p53 and the Recurrence of HCC after OLT

**Figure 5:**
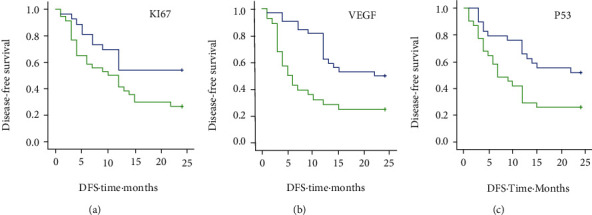
(a) Disease-free survival (DFS) of patients with negative and positive expression of Ki67 expression; blue line: negative; light green line: positive; Log Rank *P* = 0.036; Breslow *P* = 0.047; (b) disease-free survival (DFS) of patients with negative and positive expression of VEGF. Blue line: negative; light green line: positive; Log Rank *P* = 0.003; Breslow *P* = 0.001. (c) Disease-free survival (DFS) of patients with negative and positive expression of p53, Log Rank *P* = 0.015; Breslow *P* = 0.011.

Single-factor survival analysis showed that the positive expression of Ki67 (Log Rank *P* = 0.036 and Breslow *P* = 0.047), the positive expression of VEGF(*P* = 0.003 and *P* = 0.001), and the positive expression of p53 (*P* = 0.015 and *P* = 0.011) were associated with tumor recurrence, respectively (Table 3, Figures 5(a)–5(c)).

As analyzed by multivariate regression of COX, the diameter and number of tumors (*P* = 0.016), the tumor encapsulation (*P* = 0.022), the vascular invasion (*P* = 0.009), the TNM stage (*P* = 0.036), and the positive expression of VEGF (*P* = 0.005) in HCC were associated with the HCC recurrence after OLT. However, the positive expression of Ki67 (*P* = 0.142) and p53 (*P* = 0.062) did not predict the tumor recurrence and metastasis (Table 4).

## 4. Discussion

The recurrence of HCC gives rise to an unsatisfactory survival rate of HCC patients after OLT. Several studies suggested that the causes of HCC recurrence after OLT might include the following [22–25]: (1) Micrometastasis of cancer cells had occurred before transplantation. (2) Handling the diseased liver during the operation caused tumor rupture and thereby iatrogenic cancer metastasis. (3) The use of immunosuppressants promoted the proliferation and invasion of tumor cells. Recent studies showed that tumor cells and several biological molecules secreted by the microenvironment of tumors play an important role in the recurrence of tumors [26]. Preoperative detection of these molecular markers and postoperative quantitative assays of corresponding antibodies may predict tumor recurrence after OLT.

The current study showed that the expression of Ki67 in HCC was correlated with the number of tumors and the grade of tumor differentiation in HCC patients whereas it was not associated with other clinicopathological characteristics. It is suggested that the expression of Ki67 is related to the proliferation and malignant biological activities of liver cancer cells. In addition, the positive expression rate of Ki67 was higher in the recurrence group than the control group. The patients with positive expression of Ki67 had worse disease-free survival after surgery. It is suggested that HCC with high expression of Ki67 is prone to invasion and metastasis. However, COX multivariate survival analysis indicated that Ki67 had no independent predictive value for tumor recurrence after OLT. We infer that Ki67 is not specific to malignant tumors, and its expression may be affected by other factors, such as nutrient supply to cells. Therefore, Ki67 may be useful in predicting the prognosis when combined with other indicators.

VEGF is an essential factor in tumor growth, which plays a role in tumor growth and invasion, and patients prognosis [17]. The increased level of VEGF is mainly secreted by an autocrine or a paracrine measure by hepatic stellate cells and tumor cells [25]. Jeng et al. found that the expression of *VEGF* mRNA in HCC patients with the portal vein tumor was significantly higher than those without, and multivariate analysis also showed that the expression of VEGF was correlated with the portal vein thrombosis [27]. In the current study, the expression of VEGF in HCC was correlated with the diameter and number of tumors, tumor differentiation, and lymph node metastasis, which is consistent with the study by Jia et al. [28]. It is indicated that VEGF is involved in the development and progression of HCC. In addition, the positive expression rate of VEGF in the recurrence group was higher than the control group and the expression of VEGF was negatively correlated with the tumor-free survival. Therefore, VEGF is a critical risk factor for HCC recurrence after OLT and is an independent predictor of tumor recurrence after OLT in HCC patients.

In this study, p53 expression was associated with tumor encapsulation and tumor diameter, but not with the age, serum AFP level, number of tumors, tumor differentiation, and TNM stage (*P* > 0.05). It indicates that the proliferation and invasion of liver cancer cells after OLT are related to the expression of p53. Additionally, the positive expression rate of p53 was high in the recurrence group and it was associated with tumor recurrence. However, it was showed that the positive expression of p53 did not exhibit predictive value in tumor recurrence after OLT in HCC patients.

There were several limitations regarding the study. It was a retrospective study and had a relatively small sample. Only the correlationship between markers and clinicopathological characteristics were demonstrated but no in-depth mechanisms were revealed. Future studies with a larger cohort of patient samples will be needed to further support the findings.

## 5. Conclusion

In conclusion, Ki67, VEGF, and p53 are associated with the recurrence of HCC patients after OLT. Nevertheless, only VEGF independently predicts the recurrence of HCC patients after OLT. It is necessary to identify robust predictors of HCC recurrence after OLT, which facilitates the screening of patients with a high risk of HCC recurrence.

## Figures and Tables

**Table 1:** Correlation between the expression of Ki67, VEGF, and p53 and clinicopathological characteristics of HCC patients.

Clinicopathological characteristics		Ki67 expression			VEGF expression			p53 expression		
		-	+	*P*	-	+	*P*	-	+	*P*
Gender				1.000^∗^			1.000^∗^			0.238^∗^
	Male	25	32		30	27		29	28	
	Female	2	1		2	1		0	3	
Age				0.388^∗^			0.088^∗^			1.000^∗^
	≦60	22	32		31	23		26	28	
	>60	4	2		1	5		3	3	
Child-Pugh class				1.000^∗^			0.883^∗^			0.494^∗^
	A	8	11		10	9		8	11	
	B	18	12		22	18		21	19	
	C	0	1		0	1		0	1	
Tumor size				0.203^†^			0.037^†^			0.044^†^
	≦5.0	16	15		21	10		19	12	
	>5.0	10	19		11	18		10	19	
Tumor number				0.005^†^			0.005^†^			0.617^†^
	1	19	12		11	20		16	15	
	≧2	7	22		21	8		13	16	
AFP				0.174^†^			1.000^†^			0.419^†^
	>400	6	14		11	9		8	12	
	≦400	20	20		21	19		21	19	
TNM staging				0.122^∗^			0.659^∗^			0.857^∗^
	I	4	2		4	2		3	3	
	II	11	9		12	8		4	11	
	III	6	3		3	6		9	5	
	IV	5	20		13	12		13	12	
Vascular invasion				0.398^∗^			0.783^†^			0.544^†^
	Yes	4	14		6	12		7	11	
	No	22	20		26	16		22	20	
Cirrhosis				0.072^∗^			0.379^†^			0.082^∗^
	Yes	23	23		23	23		25	21	
	No	3	11		9	5		4	10	
Tumor encapsulation				0.416^†^			0.578^†^			0.022^∗^
	Yes	21	21		24	19		25	18	
	No	5	12		8	9		4	13	
Lymphatic metastasis				1.000^∗^			0.025^∗^			0.750^∗^
	Yes	1	1		0	2		0	2	
	No	25	33		32	26		29	29	
Differentiation				0.038^∗^			0.035^∗^			0.081^∗^
	Low	1	10		2	9		2	9	
	Medium	19	18		22	15		21	37	
	High	6	6		8	4		6	12	

∗: Fisher test; †: Pearson chi-square test; “+”: positive; “-”: negative.

**Table 2:** The expression of Ki67, VEGF, and p53 in the recurrence group and the control groups.

Name	Expression	Relapse group	Control group	95% CI^∗^	*P* value
Ki67	Negative	12	14	(0.288, 3.718)	0.015
	Positive	25	9		
VEGF	Negative	16	16	(0.404, 3.588)	0.008
	Positive	21	7		
p53	Negative	14	15	(0.043, 2.823)	0.035
	Positive	23	14		

∗: logistic analysis.

**Table 3:** Correlation between Ki67, VEGF, and p53 expression and tumor recurrence.

Name	*χ* ^2^	Log Rank *P* value	*χ* ^2^	Breslow *P* value
Ki67	4.404	0.036	3.936	0.047
VEGF	8.807	0.003	11.614	0.001
p53	5.947	0.015	6.445	0.011

Note: Kaplan-Meier analysis with two analytical methods.

**Table 4:** Correlation between the expression of Ki67, VEGF, and p53 and recurrence of HCC after OLT.

	*β*	S.E	Wald *χ*^2^	*P* value	OR	95% CI^∗^
Ki67	0.642	0.438	2.153	0.142	1.901	(-0.513, 1.872)
VEGF	1.387	0.492	7.942	0.005	4.004	(0.346, 3.265)
p53	0.801	0.430	3.474	0.062	2.228	(-0.147, 2.101)

∗: COX multifactor regression analysis.

## Data Availability

The data used to support the findings of this study are available from the corresponding authors upon request.
